# Exploiting the Allelopathic Potential of Aqueous Leaf Extracts of *Artemisia absinthium* and *Psidium guajava* against *Parthenium hysterophorus*, a Widespread Weed in India

**DOI:** 10.3390/plants8120552

**Published:** 2019-11-28

**Authors:** Dhriti Kapoor, Rinzim, Anupam Tiwari, Amit Sehgal, Marco Landi, Marian Brestic, Anket Sharma

**Affiliations:** 1 School of Bioengineering and Biosciences, Lovely Professional University, Delhi-Jalandhar Highway Phagwara, Punjab 144411, India; dhriti405@gmail.com (D.K.); rinzim96@gmail.com (R.); anupambhu2011@gmail.com (A.T.); amit.16824@lpu.co.in (A.S.); 2 Department of Agriculture, Food and Environment, University of Pisa, 56124 Pisa, Italy; 3 Department of Plant Physiology, Faculty of Agrobiology and Food Resources, Slovak University of Agriculture, 94976 Nitra, Slovakia; marian.brestic@uniag.sk; 4 Department of Botany and Plant Physiology, Faculty of Agrobiology, Food and Natural Resources, Czech University of Life Sciences, 16500 Prague, Czech Republic; 5 State Key Laboratory of Subtropical Silviculture, Zhejiang A&F University, Hangzhou 311300, China

**Keywords:** allelopathy, botanical herbicide, bio-herbicide, invasive plants, weeds, weed control

## Abstract

*Artemisia absinthium* and *Psidium guajava* are powerful sources of secondary metabolites, some of them with potential allelopathic activity. Both the species grow together in India with a weed (*Parthenium hysterophorus*) that is becoming extremely invasive. The aim of the present research was to test the allelopathic effect of *A. absinthium* and *P. guajava* aqueous leaf extracts on seed germination, seedling growth (shoot and root length), as well as some biochemical parameters (enzymatic and non-enzymatic antioxidants, photosynthetic pigments, osmolytes, and malondialdehyde by-products) of *P. hysterophorus* plants. Leaf extracts of both *A. absinthium* and *P. guajava* constrained the germination and seedling development (root and shoot length), affected pigment content (chlorophylls, carotenoids), stimulated the activity of antioxidant enzymes, and increased the level of malondialdehyde by-products of *P. hysterophorus* plants. Non-enzymatic antioxidants (glutathione and ascorbic acid) in *P. hysterophorus* leaves were, conversely, negatively affected by both leaf extracts tested in the present experiment. Although *A. absinthium* was more effective than *P. guajava* in impacting some biochemical parameters of *P. hysterophorus* leaves (including a higher EC50 for seed germination), *P. guajava* extract showed a higher EC50 in terms of root inhibition of *P. hysterophorus* seedlings. The present study provides the evidence that *A. absinthium* and *P. guajava* extract could be proficiently exploited as a botanical herbicide against *P. hysterophorus*.

## 1. Introduction

*Parthenium hysterophorus* is a blooming plant which belongs to the Heliantheae (Asteraceae) family. It is commonly known as congress grass, carrot weed, and broom bush in India. Similarly, in the USA the experts refer to it as feverfew and false ragweed [1]. *P. hysterophorus* is the product of natural hybridization between *P. confertum* and *P. bipinnatifidum*. *P. hysterophorus* can thrive in hostile environments and suppresses the growth of other native species due to its allelopathic effects [2]. The presence of *P. hysterophorus* decreases the stability of grazing land establishment, thereby also reducing pasture production [3]. *P. hysterophorus* has emerged as the seventh most troubling weed globally [1]. The popularity of this weed can be related to its infamous aggressive nature in the surroundings and crop fields [1], and *P. hysterophorus* infestations are increasing rapidly in many areas in India. In 1951, the *Parthenium* was first discovered in Poona (Maharashtra State). In 1972, its habitat expanded to Kerala in the south and Kashmir in the north. Later, in 1979 it expanded and advanced up to Assam. Currently, it can be found all over the subcontinent. It infests about 5 million hectares in Karnataka state, making it the most dominant weed in the area [4]. Particularly in India, *Parthenium* now thrives even in regions with prohibitive climatic conditions [5].

The dominant nature of this weed is due to its strong reproductive potential and its ability to grow at an exponential rate [1]. A bitter glycoside parthenin and SQL (sesquiterpene lactones) are the major chemical constituents of *P. hysterophorus*. All parts of *P. hysterophorus*, including trichomes and pollen, contain SQL [6]. Parthenin, ambrosin, and hymenin have been considered to be the main components of this weed responsible for its strong allelopathic effects on various crops [7,8,9]. Apart from the loss of crop yield and plant biodiversity, *P. hysterophorus* is also considered hazardous for human and animal health (e.g., it is responsible for dermatitis after contact with their leaves) [8]. In humans, swelling and itching of the mouth and nose were observed when the body was exposed to its pollens. Further, it was also noted to cause asthma (allergic bronchitis) in the later stages [10]. In the last few decades, the elimination of invasive plants has been done with the application of synthetic (chemical) herbicides (bromacil, chlorimuron ethyl, and buctril). These herbicides can cause environmental damage and also harm living organisms, including humans. In addition, the resistance of invasive herbs has been grown stronger with the misuse and abuse of chemical herbicides. Hence, the use of bio-herbicides represents a necessary advancement in weed control, in order to produce an environmentally and economically sustainable alternative. Bio-herbicides (allelopathy and allelochemicals) have been a challenge to synthetic (chemical) approaches [11].

Allelopathy is a very realistic method to control weed spread. There has been increasing interest in research on plant allelopathy to control weeds in agroecosystems [12,13]. The chemistry of allelochemicals affords control of the weeds directly or indirectly and has the potential to act as bio-herbicides [14,15]. The discovery of new weed management technologies has become inevitable to overcome the constraints of synthetic herbicide. In the light of the above, allelopathy seems to be the most practical method of weed control as it fulfils the criteria of eco-friendliness and it is already cost effective in managing several weeds [2,15].

*Artemisia absinthium* is a perennial herb belonging to the Asteraceae family and is commonly known as wormwood [16]. Absinthin, silica, thujone, anabsinthine, and tannic and resinous substances are among the main bioactive constituents of their leaves and flowers [16]. In ethnobotany, *A. absinthium* is used for its anthelmintic, antispasmodic, antiseptic, and febrifuge properties [17]. *Artemisia* spp. are also known for their allelopathic properties [18,19,20,21] especially against other species that can become invasive in some areas, such as *Convolvulus arvensis* and *Portulaca oleracea* [19].

*Psidium guajava* (guava) belongs to the Myrtaceae family and its leaves contain some bioactive compounds, like avicularin, quercetin, and guaijaverin [22]. The guava leaves also contain potential allelopathic metabolites [23], such as flavonoids, terpenoids, and cyanogenic acids [24]. Only a few studies have tested the allelopathic effects of guava against other plant species [25].

The present investigation was designed to test the allelopathic effect of aqueous leaf extracts of *A. absinthium* and *P. guajava* on seed germination, seedling growth, and some biochemical parameters of *P. hysterophorus*, thus exploring the possibility to use those extract as a bio-herbicide against this weed that is becoming extremely invasive in India.

## 2. Materials and Methods

The present work was performed in the School of Bioengineering and Biosciences Lovely Professional University, Phagwara, Punjab, India. For this experiment, *P. guajava* and *A. absinthium* were harvested in the wild from non-anthropic areas surrounding the University campus. Plants were harvested at their best balsamic period and before the flowering stage.

### 2.1. Aqueous Extract Preparation

After washing the leaves with tap water, the leaves were additionally washed with distilled water. The leaves were air dried for one month at room temperature and then ground to powder by using a mortar and pestle. The extracts were made by mixing 100 g of powdered leaves in 1000 mL of sterilized water and were kept at room temperature for 2 h. Leaf extracts were then filtered (Whatmann No. 1) and the crude extract was diluted to obtain different concentrations, i.e., 25%, 50%, and 75% (w/v) solutions. Treatments were cataloged as: P1 (25%), P2 (50%), P3 (75%), and P4 (100%) for *P. guajava* and A1 (25%), A2 (50%), A3 (75%), and A4 (100%) for *A. absinthium*. The control (CN) represents plants treated with distilled water. All the solutions were adjusted with sulfuric acid to pH 7.0 to avoid the confounding effect of different pH on plant performances.

### 2.2. Plant Material

The *P. hysterophorus* seeds were sown in mud pots measuring 10 cm in diameter and 10 cm in depth, filled with 60 g of top soil (sand/loam, 2:1). Freshly prepared concentrates of 25%, 50%, 75%, and 100% were sprayed on the surface of *Parthenium* seedlings in order to uniformly cover all the seedling surface.

After the first spray, two consecutively sprays were given on day 5 and 10. The control pots were also sprayed with distilled water. Every treatment was replicated three times. The seedlings were harvested following a month after sowing and were washed with tap water to clear any soil remaining on the roots. After that, the seedlings were examined for biophysical and biochemical parameters. 

### 2.3. Pigment Analysis

#### 2.3.1. Chlorophyll Content

A total of 1 g of fresh leaves was crushed using a mortar and pestle in 3 mL of 80% acetone. Then, the homogenized material was centrifuged at 10,000*g* for 20 min at 4 °C (Eltek cooling centrifuge, Elektrocraft Pvt. Ltd., India). The supernatant absorbance was collected at 645 and 663 nm and pigments were quantified according to Arnon [26] by using a UV-visible, double beam spectrophotometer (Systronics 2202, Systronics India Ltd., Ahmedabad, India).

#### 2.3.2. Carotenoid Content

A total of 1 g of fresh leaves was homogenized using a mortar and pestle in 4 mL of 80% acetone. Then, the homogenized material was centrifuged at 10,000*g* for 20 min at 4 °C. The supernatant absorbance was collected at 480 and 510 nm and carotenoid contents were quantified according to Maclachlan and Zalik [27].

### 2.4. Malondialdehyde (MDA) Content

Membrane damage was assessed in terms of MDA by-product content, following the method of Heath and Packer [28]. Leaves were extracted using 0.5 g of fresh material in 5 mL of 0.1% (w/v) trichloroacetic acid (TCA) and centrifuged at 5000*g* for 10 min a 4 °C. Then, 1 mL of supernatant was mixed with 6 mL of 20% (w/v) TCA, containing 0.5% (w/v) of thiobarbituric acid. This mixture was heated at 95 °C for 30 min and then cooled in an ice bath. The absorbance of the supernatant was taken at 532 nm. Correction of non-specific absorbance was done by subtraction of absorbance taken at 600 nm.

### 2.5. Proline Content

The method used by Bates et al. [29] was used for proline estimation. An aliquot of 0.5 g of fresh leaves was homogenized using a mortar and pestle in a sulfosalicylic acid solution (3% v/v), and centrifuged for 10 min at 10,000*g* for 10 min at 4 °C. Then, 2 mL of ninhydrin and glacial acetic acid were added to 2 mL of the supernatant and the mixture was incubated in a boiling water bath for 1 h. Absorbance was taken at 520 nm.

### 2.6. Glycine-Betaine (GB) Content 

The Grieve and Grattan [30] method was used to measure GB content. A 1 g amount of fresh leaves was homogenized using a mortar and pestle in 10 mL of distilled water and then the extract was filtered using filter papers (Whatman N°1). After filtration, 1 mL of the supernatant was collected and 1 mL of 2 M HCl was added. Then, 0.5 mL of this mixture were taken and 0.2 mL of potassium tri-iodide solution was added. The mixture was cooled and shaken for 90 min in an ice bath. After that, 2 mL of chilled distilled water and 20 mL of 1-2 dichloromethane was added. Two layers appeared in the mixture. The upper layer was discarded and the absorbance of the organic layer was taken at 365 nm.

### 2.7. Antioxidant Enzymes

#### 2.7.1. Catalase (CAT) Activity

The catalase activity was determined by using the method described by Aebi [31]. The decomposition rate of hydrogen peroxide was followed by a decline in absorbance at 240 nm in a reaction mixture containing 1.2 mL of hydrogen peroxide (15 mM), 300 μL of enzyme extract, and 1.5 mL phosphate buffer (50 mM; pH 7.0).

#### 2.7.2. Superoxide Dismutase (SOD) Activity

Superoxide dismutase activity was determined according to the method given by Kono [32]. In the test cuvettes, a mixture of 1.3 mL of sodium carbonate buffer (50 mM, pH 10.2), 500 μL of 24 μM nitroblue tetrazolium (NBT), and 100 μL Triton X-100 (0.03% v/v) was prepared. The reaction started after adding 100 μL hydroxylamine hydrochloride. After two minutes, 70 μL of the enzyme extract was added and the rate of NBT reduction was recorded following the rise in absorbance at 540 nm.

#### 2.7.3. Dehydroascorbate Reductase (DHAR) Activity

The DHAR activity was estimated according to the method of Dalton et al. [33]. The mixture contained 1.5 mM reduced glutathione (reduced), 50 mM of phosphate buffer. 0.2 mM dehydroascorbate, and the crude extract. Then, the absorbance was taken at 265 nm. Using the extinction coefficient 14 mM^−1^ cm^−1^, the enzyme activity was determined.

#### 2.7.4. Ascorbate Peroxidase (APX) Activity

An aliquot of 0.5 g of leaves was extracted in 3 mL of phosphate buffer (50 mM, pH 7.0) and centrifuged at 5000*g* for 20 min. A mixture of 1.5 mL phosphate buffer (50 mM, pH 7.0), 300 μL ascorbate (0.05 mM), 600 μL H_2_O_2_ (1 mM), and 600 μL of plant extract was prepared and the reduction in absorbance was monitored at 290 nm [34].

### 2.8. Total Phenolic Content 

The total phenolic content was measured according to the method given by Singleton and Rossi [35]. A total of 0.4 g of dried leaves was crushed using a mortar and pestle in 40 mL of 60% ethanol (v/v). After that, the extract was filtered using Whatman no. 1 filter paper and was diluted to 100 mL using 60% ethanol (v/v). Then, a 2.5 mL extract was taken and was diluted again with 25 mL of milli-Q water. In a 2 mL of sample extract, 10 mL of Folin–Ciocalteu reagent was added and the solution was mixed vigorously. Then, 2 mL of 75% sodium carbonate (w/v) solution was added after 5 min. The absorbance was taken at 765 nm.

### 2.9. Glutathione Content

The glutathione content in leaf samples was determined using the method described in Sedlak and Lindsay [36]. Extraction was performed as described by Sedlak and Lindsay [36]. Then, 100 mL of plant extract were added to 1 mL of Tris-HCl buffer, 4 mL of absolute methanol, and 50 μL of 5’-dithiobis-2-nitrobenzoic acid. The mixture was incubated for 50 min at room temperature and the absorbance of the supernatant was taken at 412 nm.

### 2.10. Ascorbic Acid Content 

The method in Roe and Kuether [37] was used to measure the ascorbic acid content. In 0.5 mL of plant extract, 0.5 mL of 50% TCA and 100 mg of charcoal were added. The mixture was mixed properly and then filtered with a Whatman no. 1 filter paper. Then, 0.4 mL of 2,4-dinitro phenyl hydrazine was added to the filtrate and the mixture was incubated for 3 h at 37 °C, followed by a cooling bath. Lastly, 1.6 mL of 65% H_2_SO_4_ was mixed and kept at room temperature for 30 min. Sample absorbance was taken at 520 nm.

### 2.11. Statistical Analysis

All the experiments were carried out in triplicate and performed three times independently with consistent results. A representative dataset is reported herein. Data are expressed as the mean ± SD of replicates. All data were subjected to Bartlett’s test to assess homoscedasticity of data across populations. Differences between treatments, for each parameter under study, were then evaluated using one way-analysis of variance (ANOVA) and followed by Duncan’s test (*p* ≤ 0.05). Values of EC50 for growth parameters were calculated by fitting the data to a dose-response polynomial curve. The difference in EC50 between the two extracts was analyzed using Student’s t-test (*p* ≤ 0.05). All the statistical analyses were carried out using the SPSS16 software (SPSS INC., Chicago, IL, USA). 

## 3. Results

### 3.1. Seed Germination

Both the aqueous leaf extracts of *A. absinthium* and *P. guajava* reduced the germination percentage of *P. hysterophorus* seed. There was a significant decline in seed germination by using increasing concentration of *P. guajava* extract (P1–P4), whereas A1, A2, and A3 induced a similar inhibition of seed germination (Table 1). Of note, *A. absinthium* extract showed a higher EC50 than *P. guajava* one.

### 3.2. Growth Parameters

Both extracts of *A. absinthium* and *P. guajava* inhibited the shoot and root length of *P. hysterophorus*. *A. absinthium* extract was effective even at the lowest concentration applied (25%), whereas only the highest dose of *P. guajava* inhibited significantly the root development. *P. guajava* extract showed a higher EC50 than that isolated from *A. absinthium*.

**Figure 1:**
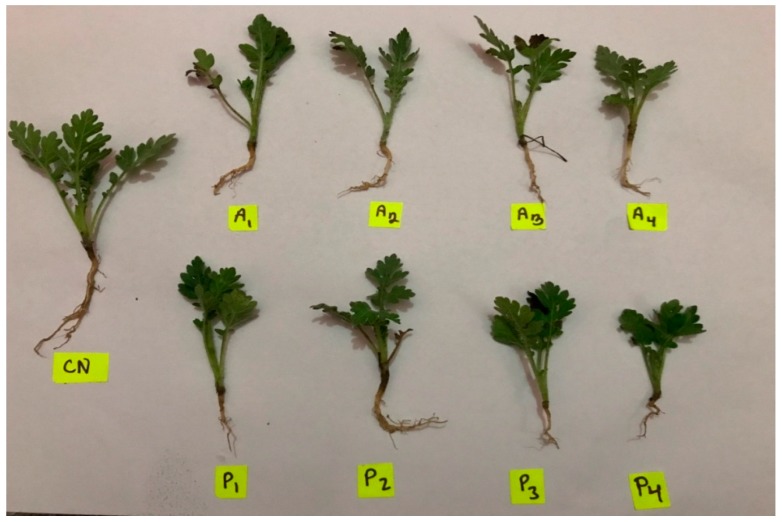
Morphological comparison of the *Parthenium* seedlings treated with different concentrations of *Artemisia absinthium* and *Psidium guajava*. (CN = Control, A1 = *Artemisia* 25%, A2 = *Artemisia* 50%, A3 = *Artemisia* 75%, A4 = *Artemisia* 100%, P1 = *Psidium* 25%, P2 = *Psidium* 50%, P3 = *Psidium* 75%, and P4 = *Psidium* 100%).

In terms of the reduction in shoot length, both of the extracts started to be effective even at the lowest dose. Minimum root and shoot lengths were recorded in seedlings treated with a higher level (100%) of both *A. absinthium* and *P. guajava* extract (Table 1, Figure 1). No differences in terms of EC50 were observed between the two tested extracts.

### 3.3. Pigments 

Chlorophyll content decreased when the leaves of *Parthenium* were treated with leaf extract of both *A. absinthium* and *P. guajava*. The most significant decrease in chlorophyll content was recorded in seedlings of *Parthenium* that were treated with the highest concentration of *P. guajava* extract (100%; P4) when compared to *A. absinthium*. Seedlings treated with a lower concentration (25%) of *P. guajava* and *A. absinthium* showed a smaller reduction in chlorophyll content (Table 2).

*Parthenium* seedlings only showed a decrease in carotenoid content when treated with the highest dose of *P. guajava* extract (P4).

### 3.4. MDA Content

The MDA content only increased significantly in seedlings of *Parthenium* when treated with 100% *P. guajava* extract. No other significant differences were observed for lower concentrations of *P. guajava* extract nor with other concentrations of *A. absinthium* (Table 3).

### 3.5. Proline and Glycine–Betaine Content

Minimum proline content was observed in seedlings treated with the lowest concentration of aqueous leaf extract of *P. guajava* (P1, 25%). No other differences were detected between treated and control plants of *P. hysterophorus* (Table 3).

Glycine-betaine content was enhanced in A3 and A4 plants as well as in P2–P4 plants, whereas A1, A2, and P1 plants showed similar glycine-betaine levels to those found in the control plants of *P. hysterophorus* (Table 3).

### 3.6. Contents of Ascorbic Acid, Glutathione, and Total Phenols

The ascorbic acid level decreased in plants treated with the highest dose of aqueous leaf extract of both *A. absinthium* and *P. guajava*, in which the minimum amount of ascorbic acid was observed (A4 and P4 treated seedlings). The maximum ascorbic acid was recorded in control plants (Table 4).

When *Parthenium* seedlings were sprayed with different concentrations of aqueous leaf extract of *A. absinthium* and *P. guajava*, the seedlings of *Parthenium* exhibited a significant drop in glutathione content. The minimum glutathione content was found in seedlings treated with 100% extract of *P. guajava* (Table 4).

Seedlings of *Parthenium* treated with different concentration of *A. absinthium* and *P. guajava* showed enhanced total phenolic content compared to untreated seedlings. The maximum increase in total phenolic content was recorded in P3 and P4 plants (Table 4) and in A3 and A4 plants, with the former showing the highest values among the treatments and species.

### 3.7. Antioxidative Enzymes

Activity of CAT was enhanced by all of the treatments with both the extracts of *A. absinthium* and *P. guajava*. The maximum CAT activity was observed in plants treated with the highest concentrations (A4 and P4) of both *A. absinthium and P. guajava*. (Table 5).

Enhanced activity of SOD was observed in seedlings treated with different concentrations of aqueous leaf extract of *A. absinthium* and *P. guajava*. Maximum activity of SOD was observed in A3 and A4, as well as in P4 plants (Table 5).

APX activity was stimulated in seedlings of *Parthenium* treated with 75% and 100% of *A. absinthium* and 100% of *P. guajava* leaf extracts. DHAR increased similarly to APX in A3 and A4 plants, whereas all the treatment with *P. guajava* extract promoted the increase in DHAR activity. (Table 5).

## 4. Discussion

The biological control of weeds using allelopathic species or the use of allelochemicals isolated from plant extracts is preferred over both mechanical and chemical control in agriculture. Mechanical control results in high costs of management whereas the chemical control poses serious concerns for human health as well as environmental safety. Furthermore, intensive chemical control also promotes the development of herbicide-resistant weeds [3].

The current study showed that the aqueous leaf extracts of *A. absinthium* and *P. guajava* were effective in limiting the seed germination and the growth of *P. hysterophorus*, which is becoming invasive in India as well as in other areas of the world. The aqueous leaf extracts of *A. absinthium* and *P. guajava* had a critical impact on seed germination and seedling development (shoot and root length). As the concentration levels increased, these impacts likewise increased. To prove the interest in controlling this weed with other bio-herbicides, the phytotoxic effects of other allelopathic grasses, like *Dichanthium annulatum*, *Cenchrus pennisetiformis*, and *Sorghum halepense*, have also been reported against *Parthenium* [38].

The range of concentration both *A. absinthium* and *P. guajava* leaf extracts tested herein (25–100%) affected either the germination percentage or the root and shoot length of *Parthenium*. Notably, besides a different chemical composition of leaf extract between the two species tested herein (not characterized in the present experiment), aqueous extracts obtained from both of the species exerted similar negative effect in terms of seed germination and root and shoot development of *P. hysterophorus* when applied at the highest concentration level. *A. absinthium* had a higher EC50 in terms of seed germination inhibition, whereas *P. guajava* showed a significantly higher EC50 in terms of root inhibition. Therefore, these two extracts could be used together or individually at different stages during *P. hysterophorus* infestations. According to other research [38,39,40], the aqueous extracts of allelopathic grasses can be efficiently exploited to control the infestation of *P. hysterophorus* in the field due to their capacity to affect different pathways of this weed.

Chlorophylls and carotenoids are key photosynthetic pigments for plants, and their content and functionality are essential to absorb and direct the light to photosystems [41]. In this investigation, it was observed that the aqueous leaf extracts of *A. absinthium* and *P. guajava* reduced the level of chlorophylls and carotenoids, which suggests possible photosynthetic limitations exerted by both the extracts to *Parthenium* leaves. An impaired photosynthetic process can generate a surplus of excitation energy burden in the chloroplast, thus leading, in turn, to an overproduction of harmful reactive oxygen species (ROS) [42]. Increased levels of MDA by-products observed in *Parthenium* seedlings support the production of free radicals and the occurrence of lipid peroxidation events [43] induced by the treatment with *A. absinthium* and *P. guajava* leaf extracts.

Ascorbic acid (ASA) and glutathione (GSH) are considered to be key components of non-enzymatic cellular antioxidant defense system [44]. For ASA to act as an antioxidant, it is necessary to preserve its reduced form by the activity of DHAR [44]. In this study, it was observed that ASA content was remarkably reduced with increases in the concentrations of *A. absinthium* and *P. guajava* extracts. This occurred in concomitance with an enhancement in DHAR (compared to the control plants), which suggests that the regeneration of ASA in its fully reduced form was not enough to preserve the ASA pool. The content of glutathione was also drastically reduced by *A. absinthium* and *P. guajava* extracts application over the *Parthenium* leaves. The stimulation of other non-enzymatic antioxidants, such as glycine-betaine and total phenolic compounds, might have further represented an attempt by *Parthenium* plants to counteract the oxidative stress induced by the application of *A. absinthium* and *P. guajava* extracts.

Besides DHAR, other antioxidant enzymes act as ROS scavengers in plant cell, including SOD, CAT, and APX [45]. The present experiment revealed that the activity of all these enzymes was stimulated by the treatment of *Parthenium* plants with *A. absinthium* and *P. guajava* extracts. Higher levels of MDA in cells subjected to these allelochemicals suggested that the antioxidant enzymatic system, although enhanced, did not completely eliminate the surplus of ROS and did not protect from ROS-triggered oxidative insult. A huge body of experimental evidence suggests that the induction of oxidative stress and enhancement of antioxidant battery are common responses exhibited by different plant species to allelochemicals (for a review see [46]). Although the precise molecular target of ROS generated in plants responding to allelochemicals is not fully recognized, there is no doubt that several allelochemicals act as prooxidants.

Allelochemicals are well-known inhibitors of germination and plant growth and, in most cases, they lead to the modification of cell redox status [46]. With the data provided by our dataset, we were not able to find the connection between the ROS triggered by *A. absinthium* and *P. guajava* application and the reduction in root and shoot development in *Parthenium* plants. It seems, however, possible that the mechanical properties of the cell wall may be modified by enzymes, ROS, and their interaction [47]. Among cell wall proteins, endoglucanases, xyloglucan endotransglycosylases, pectinases, pectin esterases, debranching enzymes, and non-enzymatic proteins, such as expansins, are responsible for cell wall extensibility [48]. The second group of agents affecting cell wall extensibility are ROS, derived by spontaneous reaction or produced/consumed by cell wall associated proteins, such as APX, NADPH oxidase, and SOD [46]. Therefore, both the direct effect of some ROS (principally ^•^ OH and H_2_O_2_; ^•^ OH which can be produced by Fenton reactions from NADPH oxidase-derived O_2_^•^− or by peroxidases supplied with O_2_ and NADH [49]) and their inaction with the abovementioned cell wall protein might responsible for the reduction in plant growth. However, other possible effects exerted by both the tested extracts (e.g., hormonal interference, etc.) might have additionally contributed to the limited root and shoot growth inhibition of *Parthenium* plants and this poses the bases for future research.

## 5. Conclusions

This investigation revealed that both the aqueous leaf extracts of *Artemisia absinthium* and *Psidium guajava* have a significant impact on germination and seedling development (root and shoot length) of the widely spread weed *Parthenium hysterophorus*. Both the aqueous extract of *Artemisia absinthium* and *Psidium guajava* also affected the photosynthetic pigments (chlorophylls and carotenoids) of *Parthenium* plants. The level of non-enzymatic antioxidants (GSH, ascorbic acid) were also significantly reduced in *Parthenium* plants treated with both aqueous extracts of *A. absinthium* and *P. guajava.* Conversely, the antioxidants enzymes (SOD, DHAR, APOX, CAT) and total phenolic content were enhanced in *Parthenium* plants treated with aqueous leaf extracts of *A. absinthium* and *P. guajava*. Higher levels of peroxidation (malondialdehyde by-products) in cells subjected to both the plant extracts suggest that the antioxidant enzymatic system, although induced, did not efficiently counteract reactive oxygen species overproduction. In conclusion, even though the mechanism by which both the extracts inhibit the growth of *P. hysterophorus* plants is not completely clear and needs further investigation, it is remarkable that the aqueous extracts of both *A. absinthium* and *Psidium guajava* can be exploited as botanical herbicides to control the spread of *P. hysterophorus*.

## Figures and Tables

**Table 1:** Allelopathic effects of various concentrations of leaf extract of *Artemisia absinthium* and *Psidium guajava* on seed germination and root and shoot length of *Parthenium hysterophorus* seedlings.

Treatment	Seed Germination (%)	Root Length (cm)	Shoot Length (cm)
CN	81.38 ± 9.02 ^a^	13.46 ± 1.61 ^a^	23.4 ± 1.11 ^a^
A1	55.42 ± 7.72 ^bc^	5.53 ± 0.92 ^b^	17.7 ± 1.08 ^c^
A2	53.53 ± 10.4 ^bc^	5.23 ± 1.00 ^bc^	15.2 ±1.05 ^d^
A3	45.36 ± 5.35 ^cd^	4.77 ± 1.02 ^bc^	14.7 ± 1.35 ^d^
A4	36.00 ± 6.76 ^de^	3.6 ± 0.69 ^c^	11.9 ± 1.62 ^e^
P1	63.72 ± 5.66 ^b^	6.36 ± 1.02 ^b^	20.4 ± 0.87 ^b^
P2	47.62 ± 8.07 ^cd^	6.5 ± 0.65 ^b^	17.4 ± 1.17 ^c^
P3	35.91 ± 5.72 ^de^	4.77 ± 1.01 ^bc^	14.4 ± 0.73 ^d^
P4	28.6 ± 9.65 ^e^	3.44 ± 0.69 ^c^	10.17 ± 1.01 ^e^
P_EC50_	69.1± 0.2 B	38.0 ± 0.8 A	96.3 ± 1.0 A
A_EC50_	97.7 ± 0.4 A	24.3 ± 1.2 B	95.8 ± 1.5 A

(CN = Control, A1 = *Artemisia absinthium* 25%, A2 = *A. absinthium* 50%, A3 = *A. absinthium* 75%, A4 = *A. absinthium* 100%, P1 = *Psidium guajava* 25%, P2 = *P. guajava* 50%, P3 = *P. guajava* 75%, and P4 = *P. guajava* 100%). Data shown here are mean ± SD and mean values with same letters are not significantly different from each other at *p* < 0.05. EC50 of both extracts were compared by Student’s t-test (*p* ≤ 0.05).

**Table 2:** Comparison of total chlorophyll and carotenoid content in seedlings of *Parthenium hysterophorus* treated with leaf extract of *Artemisia absinthium* and *Psidium guajava*.

Treatment	Total Chlorophylls Content (mg/g FW)	Carotenoids (mg/g FW)
CN	0.081 ± 0.001 ^a^	0.0151 ± 0.001 ^a^
A1	0.066 ± 0.002 ^ab^	0.0141 ± 0.0009 ^a^
A2	0.064 ± 0.0009 ^b^	0.0131 ± 0.0007 ^a^
A3	0.057 ± 0.002 ^bc^	0.0140 ± 0.001 ^a^
A4	0.055 ± 0.0006 ^bc^	0.0132 ± 0.0010 ^a^
P1	0.063 ± 0.0025 ^b^	0.0139 ± 0.0009 ^a^
P2	0.042 ± 0.0006 ^cd^	0.0127 ± 0.0010 ^a^
P3	0.033 ± 0.001 ^d^	0.0135 ± 0.0007 ^a^
P4	0.031 ± 0.002 ^d^	0.0099 ± 0.0047 ^b^

(CN = Control, A1 = *Artemisia absinthium* 25%, A2 = *A. absinthium* 50%, A3 = *A. absinthium* 75%, A4 = *A. absinthium* 100%, P1 = *Psidium guajava* 25%, P2 = *P. guajava* 50%, P3 = *P. guajava* 75%, and P4 = *P. guajava* 100%). Data shown here are mean ± SD and mean values with same letters are not significantly different from each other at *p* < 0.05.

**Table 3:** Effect on the contents of malondialdehyde (MDA), proline, and glycine-betaine in seedlings of *Parthenium hysterophorus* treated with different concentration of *Artemisia absinthium* and *Psidium guajava.*

Treatment	MDA (mg/g FW)	Proline (mg/g FW)	Glycine-Betaine (mg/g FW)
CN	1.33 ± 0.11 ^c^	1.49 ± 0.15 ^a^	0.41 ± 0.09 ^b^
A1	1.51 ± 0.09 ^bc^	1.51 ± 0.09 ^a^	0.53 ± 0.08 ^ab^
A2	1.54 ± 0.06 ^bc^	1.52 ± 0.09 ^a^	0.55 ± 0.05 ^ab^
A3	1.63 ± 0.06 ^bc^	1.53 ± 0.08 ^a^	0.56 ± 0.06 ^a^
A4	2.32 ± 0.57 ^a^	1.55 ± 0.11 ^a^	0.62 ± 0.08 ^a^
P1	1.35 ± 0.14 ^c^	1.22 ± 0.11 ^b^	0.54 ± 0.06 ^ab^
P2	1.38 ± 0.10 ^c^	1.51 ± 0.19 ^a^	0.59 ± 0.10 ^a^
P3	1.61 ± 0.10 ^bc^	1.57 ± 0.11 ^a^	0.65 ± 0.05 ^a^
P4	1.83 ± 0.13 ^b^	1.65 ± 0.15 ^a^	0.67 ± 0.06 ^a^

(CN = Control, A1 = *Artemisia absinthium* 25%, A2 = *A. absinthium* 50%, A3 = *A. absinthium* 75%, A4 = *A. absinthium* 100%, P1 = *Psidium guajava* 25%, P2 = *P. guajava* 50%, P3 = *P. guajava* 75%, and P4 = *P. guajava* 100%). Data shown here are mean ± SD and mean values with same letters are not significantly different from each other at *p* < 0.05.

**Table 4:** Comparison of ascorbic acid, glutathione, and phenolic contents in seedlings of *Parthenium hysterophorus* treated with leaf extract of *Artemisia absinthium* and *Psidium guajava*.

Treatment	Ascorbic Acid (mg/g FW)	Glutathione (mg/g FW)	Phenolic Content (mg/g FW)
CN	1.66 ± 0.12 ^a^	3.28 ± 0.15 ^a^	1.13 ± 0.1 ^f^
A1	1.55 ± 0.11 ^ab^	2.53 ± 0.19 ^b^	1.30 ± 0.11 ^de^
A2	1.45 ± 0.09 ^bcd^	2.16 ± 0.18 ^bc^	1.45 ± 0.12 ^cd^
A3	1.53 ± 0.10 ^abc^	2.30 ± 0.50 ^bc^	1.72 ± 0.10 ^b^
A4	1.34 ± 0.09 ^cd^	2.05 ± 0.05 ^c^	1.73 ± 0.10 ^b^
P1	1.52 ± 0.09 ^abc^	2.50 ± 0.10 ^b^	1.22 ± 0.05 ^ef^
P2	1.37 ± 0.10 ^bcd^	2.26 ± 0.14 ^bc^	1.53 ± 0.10 ^c^
P3	1.39 ± 0.09 ^bcd^	1.96 ± 0.07 ^c^	1.92 ± 0.09 ^a^
P4	1.31 ± 0.13 ^d^	1.43 ± 0.10 ^d^	2.04 ± 0.07 ^a^

(CN = Control, A1 = *Artemisia absinthium* 25%, A2 = *A. absinthium* 50%, A3 = *A. absinthium* 75%, A4 = *A. absinthium* 100%, P1 = *Psidium guajava* 25%, P2 = *P. guajava* 50%, P3 = *P. guajava* 75%, and P4 = *P. guajava* 100%). Data shown here are mean ± SD and mean values with same letters are not significantly different from each other at *p* < 0.05.

**Table 5:** Comparison of enzymatic activities in seedlings of *Parthenium hysterophorus* treated with different concentration of *Artemisia absinthium* and *Psidium guajava* leaf extract.

Treatment	CAT (UA/g Protein)	SOD (UA/g Protein)	APX (UA/g Protein)	DHAR (UA/g Protein)
CN	3.02 ± 0.01 ^i^	3.02 ± 0.02 ^d^	0.0131 ± 0.001 ^c^	0.006 ± 0.001 ^f^
A1	6.95 ± 0.06 ^f^	6.65 ± 0.57 ^c^	0.0132 ± 0.001 ^c^	0.007 ± 0.001 ^f^
A2	7.93 ± 0.10 ^e^	7.96 ± 0.97 ^b^	0.014 ± 0.001 ^c^	0.01 ± 0.0001 ^d^
A3	9.58 ± 0.15 ^c^	9.54 ± 0.50 ^a^	0.015 ± 0.002 ^c^	0.015± 0.001 ^c^
A4	9.84 ± 0.11 ^b^	9.66 ± 0.60 ^a^	0.020±0.0001 ^b^	0.021 ± 0.001 ^b^
P1	5.23 ± 0.11 ^h^	5.58 ± 0.80 ^c^	0.014 ±0.001 ^c^	0.008 ± 0.0009 ^e^
P2	6.6 ± 0.11 ^g^	6.53 ± 0.50 ^c^	0.016 ± 0.001 ^c^	0.013± 0.001 ^d^
P3	8.3 ± 0.10 ^d^	8.44 ± 0.50 ^b^	0.019 ± 0.001 ^b^	0.012 ± 0.001 ^d^
P4	10.2 ± 0.16 ^a^	10.3 ± 0.46 ^a^	0.027 ± 0.004 ^a^	0.024 ± 0.001 ^a^

(CN = Control, A1 = *Artemisia absinthium* 25%, A2 = *A. absinthium* 50%, A3 = *A. absinthium* 75%, A4 = *A. absinthium* 100%, P1 = *Psidium guajava* 25%, P2 = *P. guajava* 50%, P3 = *P. guajava* 75%, and P4 = *P. guajava* 100%). Data shown here are mean ± SD and mean values with same letters are not significantly different from each other at *p* < 0.05. Abbreviations: catalase, CAT; superoxide dismutase, SOD; ascorbic acid peroxidase, APX; dehydroascorbate reductase, DHAR.
